# LASS2 inhibits growth and invasion of bladder cancer by regulating ATPase activity

**DOI:** 10.3892/ol.2022.13188

**Published:** 2022-01-04

**Authors:** Haifeng Wang, Yigang Zuo, Mingxia Ding, Changxing Ke, Ruping Yan, Hui Zhan, Jingyu Liu, Wei Wang, Ning Li, Jiansong Wang

Oncol Lett 13: 661-668, 2017; DOI: 10.3892/ol.2016.5514

Subsequently to the publication of the above article, an interested reader drew to the authors’ attention that the Matrigel invasion assays in Fig. 2C on p. 664 appeared to contain an error: Essentially, the T24 cell data were mistakenly selected twice for the si-LASS2 group, and also used to represent the RT4 experiment. However, the authors have consulted their original data, and have identified the correct data panel for the RT4 / si-LASS2 experiment.

The corrected version of Fig. 2, showing the correct data for Fig. 2C, is shown on the next page. Note that the errors in this figure did not affect either the results or the conclusions reported in this study. The authors are grateful to the Editor of *Oncology Letters* for granting them the opportunity to publish this corrigendum, and regret any inconvenience caused to the readership of the Journal.

## Figures and Tables

**Figure 2.**
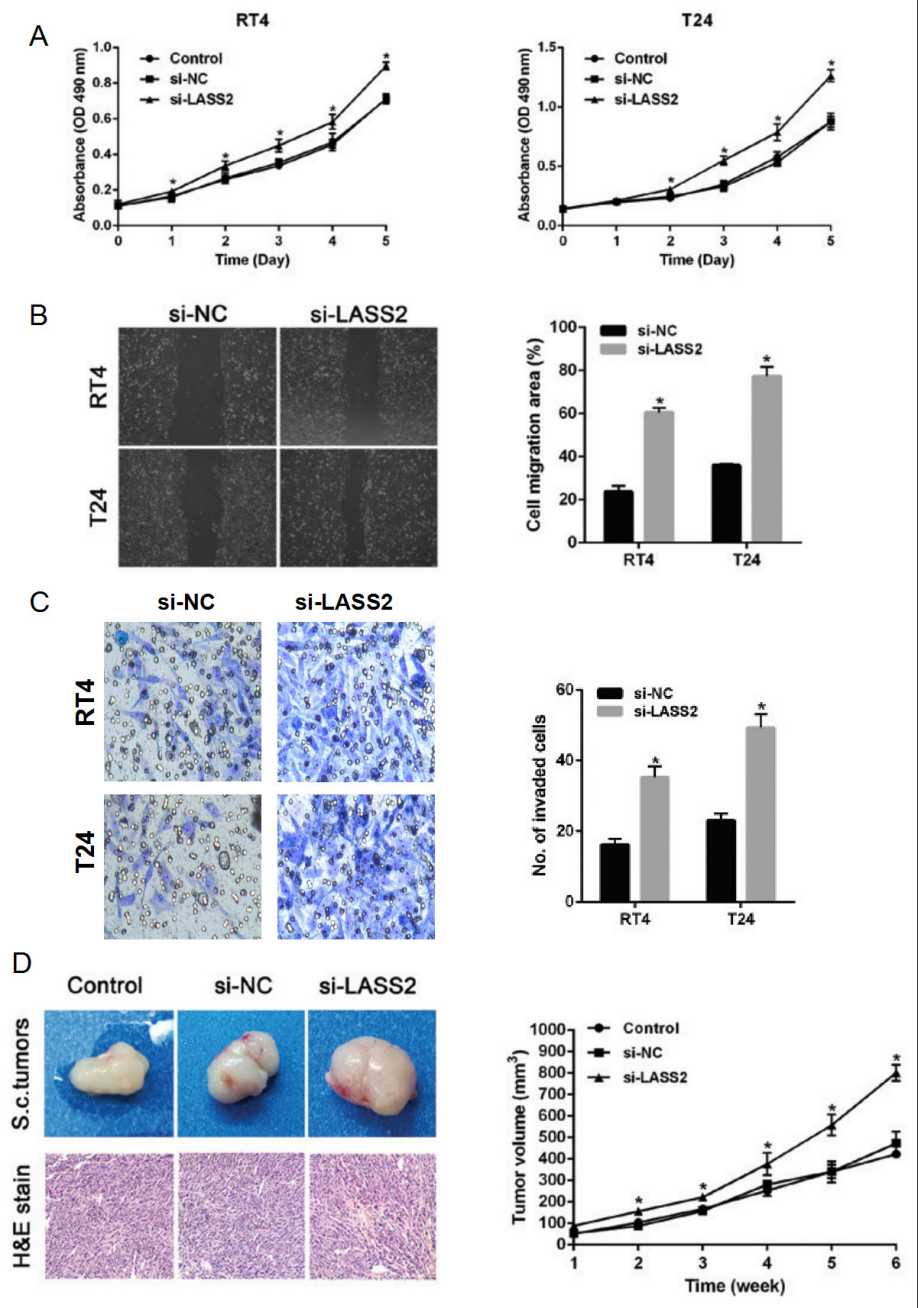
si-LASS2 exhibits cancer promoting effects *in vitro* and *in vivo*. (A) MTT assays were used to asses the viability of RT4 and T24 cells transfected with si-LASS2 or si-NC. (B) Cell migration was measured using scratch assays (×40 magnification). (C) Matrigel invasion assays were used to evaluate the invasion activity of bladder cancer cells transfected with si-LASS2 or si-NC (×100 magnification). (D) Tumor outgrowth in RT4 ×enograft mice (×40 magnification). Top images, representative tumors from the three groups after 6 weeks; bottom images, dissected tumors stained with H&E. Data are presented as the mean ± standard deviation of three independent experiments. *P<0.05 vs. si-NC group. si-LASS2, small interfering RNA targeting longevity assurance homolog 2 of yeast LAG1; si-NC, unspecific control small interfering RNA; H&E, hematoxylin & eosin.

